# Hospital and Institutional News

**Published:** 1913-02-22

**Authors:** 


### HOSPITAL AND INSTITUTIONAL NEWS.

### THE PATIENTS AND THE HOSPITALS.

Some correspondence we published last week, with that in our present issue, gives an inkling of the views and feelings of some of the patients who frequent the Voluntary Hospitals and Poor-Law Infirmaries in London. In this connection we would direct the attention of hospital managers to the paper on Poor-Law Infirmaries and Voluntary Hospitals by Dr. Thackray Parsons, and to that on the London Hospital Saturday Fund and the National Insurance Act, which contains the views and fears of the Saturday Fund Executive. We are of opinion that amongst these varying aspects of this question there are misapprehensions, and especially in regard to patients being refused treatment at Voluntary Hospitals without medical examination. We hope it may prove helpful, therefore, to open our columns to a correspondence on the questions thus raised, and that it may remove every possible ground for misapprehension on the part of all the interests involved. We rely upon those who know the facts and the Voluntary Hospitals and Infirmaries' view to write fully and freely upon the position as it exists at the moment.

### THE TAXATION OF CHARITABLE BEQUESTS.

We have elsewhere in this issue pressed the case for the remission of the legacy duty upon bequests to hospitals, a matter which has been more than once influentially recommended to the Treasury and the Chancellor. What prospect there is of so desirable a reform being brought about we do not know; but the present is an excellent time for agitating the matter, because the hospitals are being expected to relieve the State and the Government of those obligations which the Insurance Act fails to fulfil. If the moral justice of the claim fails to impress the authorities, perhaps some more practical illustration of the unwisdom of the present arrangement may have more point. We have already alluded to extensive gifts made to hospitals by generous and wealthy people during their lifetime in place of bequests which take effect only after death and are then taxed by the Revenue. By this plan the State loses the whole of the duty on the sums so given, and also in many cases actually loses on the remainder of the estate, which may thus be reduced to a size upon which a lower scale of death duties are payable. We note that last week the honorary freedom of the City of Chester was conferred upon Mr. Albert Wood, of Conway, by the Town Council of Chester in recognition of a gift of over £30,000 to the Building Fund of the Chester Infirmary. In reply to the presentation, Mr. Wood dwelt upon the reason for his generosity, explaining that the result would be to save £2,500 in duties, which the Treasury would have received had he willed the money to the infirmary instead of giving it. He expressed very strongly the opinion that charitable bequests should not be taxed. Whatever difficulty there may be in deciding the exact definition of a charity, there is surely no doubt that voluntary hospitals come within it.

### EXAMPLES OF BOTH METHODS OF HELPING HOSPITALS.

Dover is as fortunate as Chester in the matter of hospital benefaction, and has, we are glad to see, been equally grateful. The Corporation a few days ago decided to confer the freedom of the Borough upon Mr. Arthur Burr. It is true that this honour has been earned partly by his services in developing the local colliery, of which high hopes are entertained; and by his activity in promoting the erection of model villages for the workpeople and in finding work for the local unemployed. But it is also true that Mr. Burr has been a supporter of the Dover Hospital on a very generous scale; indeed a speaker on the resolution stated that his donations totalled some thousands of pounds. On the other hand, the late Mr. P. M. Coats, of Paisley, who had left £5,000 to two local hospitals in his will, revoked both bequests by a codicil dated last September " owing to the nature of recent Socialistic legislation by the present Liberal Government." We fancy that Mr. Coats was himself a Liberal in politics, and, if that was so, the remark in question acquires added significance.

### SOME LONDON STAFF APPOINTMENTS.

At King's College Hospital Dr. Robert Knox has been appointed chief radiographer. Dr. Knox is already holding similar posts at the Great Northern and the Cancer Hospitals; and at the latter he has lately had control of the entirely new building and the process of equipment with the most modern apparatus, which were completed last year. This experience should stand him in good stead at Denmark Hill, where the question of choosing the apparatus for the radiological department of the new King's College Hospital must shortly be the subject of very careful attention under expert advice. At the Cancer Hospital Dr. Knox designed several of the most useful appliances himself, and in his hands it may be confidently expected that the installation at King's will be both economical and at the same time fully complete. At University College Hospital the out-patient department for those suffering from mental disorders has been placed in the charge of Dr. Bernard Hart, who already lectures on mental diseases in the medical school. At the Westminster Hospital Dr. O. K. Williamson has been appointed assistant physician, a similar position to that which he already holds at the City of London Hospital for Diseases of the Chest.

### THE COUNTY OF LONDON INSURANCE COMMITTEE'S MEDICAL MEMBERS.

Friday, February 21, is the last day for sending in the ballot-papers which have been sent to every qualified practitioner whose address appears in "The Medical Register" as being within the County of London; and the result of the election will be watched with a somewhat curious interest. In passing we may note that neither the ballot-paper nor the accompanying letter of instructions contains any mention whatever of the number of candidates who are to be elected. Inasmuch as some sort of proportional representation by a system of preferential voting is being attempted, this omission seems to us evidence of gross carelessness in making the arrangements; unless it is really an artful device for outflanking the "last-ditchers," who would naturally get a good many "plumpers" if it were made plain that any other method of voting might let in less energetic champions of the cause that was lately upheld by the British Medical Association. The list of candidates, too, is an interesting one. There are twenty-five of them anxious for this unremunerated task, most of whom are general practitioners of no prominence outside the limited area of their own immediate practice. Mr. E. B. Turner is the outstanding exception to this statement. Then there are an Indian doctor, an anæsthetist, a statistician, a homœopath, and an Austrian pathologist. The last-named is the Dr. Ludwig Freyberger whose employment by the late Mr. J. Troutbeck, in preference to native experts in *post-mortem* pathology, created a lot of ill-feeling and friction in the ranks of the London profession a few years ago. We miss entirely any representative of the Cavendish Square region among the twenty-five.

### POLITICS FIRST, THE PEOPLE LAST.

The present Administration, or some of its most influential members, are adepts at the art of exercising administrative functions in the interests of their own party. They seem quite careless whether or no they inflict hardship and injustice on the people whom they profess to serve. In several Government departments there have been repeated instances of this; and the Insurance Commission is following the same bad path. We have already alluded to their persistent efforts to suppress Form Med. 21, and thereby to prevent insured persons exercising the rights expressly provided for them in the Act of really getting "free choice" of doctor. There can be no doubt whatever that the tyrannical system of which this forms part is designed solely in order that Mr. George may assure Parliament that the panel system is entirely successful. In London every effort is being made by the Committee, of which a Government M.P. is chairman, to induce those who have applied for permission to "make their own arrangements for medical benefit" to withdraw their applications. Every possible difficulty and discouragement, even to the printing of a special circular, is being created for this purpose. Incidentally we may state that in London many quite reasonably competent men have lately joined the panels in those districts where the first list was admittedly wholly inadequate. It is not too much to say that in the residential parts of the town the lists consisted very largely of scallywags and failures; and even now the proportion of reasonably efficient practitioners "on the panel" is much smaller than it ought to be. Such is the result of putting politics before common decency and common humanity.

### DOMICILIARY TREATMENT OF TUBERCULOSIS AMONG THE INSURED.

A point which has not so far received very much consideration arises out of the arrangements made by the sanatorium benefit sub-committee of the Brighton Insurance Committee. It will be remembered that under the panel scheme a further sixpence in respect of every insured person is provided for the domiciliary treatment of the tuberculous insured for whom sanatorium treatment is not provided or is considered inadvisable. This extra sixpence was part of the bait dangled before the doctors last November by Mr. Lloyd George; but it is now beginning to be clear that they will in reality get only a part of it. At Brighton a scheme is suggested, and will probably be adopted, whereby three-seventeenths of the fund thus provided will go in the purchase of medicines. Further arrangements are being made for the Queen's Nurses' Association to undertake the nursing arrangements in return for payments according to the work done. It is not yet definitely stated whether this money, too, comes out of the doctors' sixpences; if it does, the latter may very easily find themselves in receipt of the handsome salary of nothing at all as regards the case of their tuberculous insured patients.

### MORE INSURANCE MUDDLES.

In spite of the official enthusiasm and optimism of Mr. Masterman in the House of Commons, it is every day more apparent that the Insurance Act and everything connected with it is in a hopeless state of muddle and confusion. On Monday last the widow of a Lambeth sawyer sued Mr. J. A. Dawes, M.P., and the Insurance Commissioners for damages on the ground that there had been negligent delay in the administration of tuberculosis benefits to which he was entitled under the Act. The Judge disallowed the action against the Commissioners, but allowed the case against Mr. Dawes—the Chairman of the London Insurance Committee—to go to the jury. It is clear from the evidence that a lot of formality is necessary before a tuberculous insured person gets his benefits; and in this particular case the delays certainly seemed to be unconscionable and inexplicable, except through someone's blundering. Eventually the jury found the London Insurance Committee guilty of negligent default, but that the negligence was not the cause of death. They awarded the widow £150 damages, but the Judge entered a verdict for the defendants on the second of their findings. The question which neither Mr. Dawes nor anyone else seemed able to give a satisfactory answer to is, "Why was this man not inspected by a representative of the Committee until October 4, when his case was notified to them as being probably consumption upon August 21?"

### SOME LONDON EDITORS AND THE VOLUNTARY HOSPITALS.

The position of the editor of a London daily paper does not diminish in difficulties or pressure as the years pass. The material daily received in the editorial offices must not infrequently amount to sufficient copy to occupy three times the available space. The foreign news alone nowadays is sometimes great, and at the present time, when Parliament is paralysed and discredited, British readers are displaying greater interest in foreign politics than they do in home matters. It is interesting to note the Scylla and Charybdis of the London editors by studying their columns. On the one hand, the conscientious editor provides evidence that everything of importance is adequately noted, whilst his more sensational colleague sits at his desk and lifts trivialities to a position of importance by the use of head-lines and large type, whilst he dwarfs the news to the verge of exclusion. The first editor is apt wrongly to be charged with dulness, and the second editor, with equal injustice, held to be a man of enterprise who understands the public—what a condemnation of his readers if he does! The first editor may claim to be a follower of John Delane, and we were impressed with the excellence of *The Times* in his day by having recently to refer to its columns at that date. Representing as we do the hospitals and philanthropic institutions of these islands, we regret very much the present policy of *The Times*, which seems to be to cut down its reports on such matters to a few lines, and to destroy much of its interest and influence as a newspaper by discouraging correspondence on these subjects. Were it not for the *Daily Telegraph* and the *Morning Post* the managers of our charities might well grow discouraged to the breaking-point. The voluntary hospitals owe much to these papers, and especially to the *Daily Telegraph*, which has fought their battle in the last few months with hopeful energy. The space the *Daily Telegraph* gives to these subjects was convincingly shown by the reports of recent important meetings in connection with the Hospital Sunday and Hospital Saturday Funds. We are encouraged to hope that *The Times* may revert to John Delane's methods, so far as the voluntary hospitals are concerned at any rate, from the increased attention they have been giving lately to the effect of the Insurance Act upon the voluntary hospitals.

### THE PARSON AND THE FEVER HOSPITAL.

"As I have been refused admittance to the Calverley Hospital, it will be no use asking me to visit any children that may have the misfortune to get fever and go there." Thus writes the Rev. Arthur Whyte, curate of Rodley, near Leeds, in a letter to his parishioners. It is the outcome of a quarrel between the minister and the board of the hospital, which began during an epidemic of scarlet fever, when the curate told the board that they were sending out children before they were in fit condition, and that new cases were largely the result of contact with such children. "The hospital board," says Mr. Whyte in an interview, "then abused me, saying I had taken possession of the hospital without permission, and gone into the wards without the regulation over-all." He says that, as a matter of fact, he used the doctor's over-all. Since that time the agitation has proceeded vigorously, and similar charges have been made by two other gentlemen, Messrs. A. Guy and W. H. Sharp, both members of the hospital board. Now, announces Mr. Whyte, admission has been refused to clergymen on the plea that they carry away infection, "yet they have a cat in the wards, which runs about and carries infection anywhere." Mr. Whyte says this is not a serious thing for the clergymen themselves, as they do not go for pleasure; but it deprives the population of the advantage of sending the minister to provide a link between parents and children, only one person thus going instead of many. He says the parents appreciate the reports conveyed by the clergymen, and the system reduces the chances of carrying away infection. Mr. Whyte has made this matter public by means of the *Yorkshire Evening Post*. The hospital authorities have not so far taken up the challenge thus put forth; it is quite possible that their version of the facts may be different from Mr. Whyte's, but at least they ought to make it public.

### DEATH OF SIR THOMAS CHAVASSE.

Only a few days ago Sir Thomas Frederick Chavasse was the recipient of a presentation on the occasion of his retirement from practice and from the service of the Birmingham hospitals, which he has so long served. Still middle-aged, as men are counted nowadays, it seemed as if Sir Thomas had many years of leisure and health before him. Hunting represented to him one of the enjoyments of life which retirement rendered easy. Unfortunately he took a fall and broke his femur; and this accident has directly led to his death. Sir Thomas Chavasse was professionally educated at Edinburgh, Dublin, and Birmingham. He was lately senior surgeon to the Birmingham General Hospital, and consulting surgeon to several cottage hospitals in the district. He was M.D. (Edin.), and F.R.C.S. Edin. and England. His premature decease, with that of Mr. Eales, recorded in our columns last week, robs Birmingham of two of the most efficient and respected of the senior consultants there. As in the case of Sampson Gamgee, another famous Birmingham surgeon, Sir Thomas's fatal illness appears to have begun with an accident to his leg.

### SCHOOL CLINICS IN SURREY.

In connection with the triennial election of County Councillors in Surrey a few weeks hence there is a burning issue in the shape of a proposal touching the health of the school children in the county, it being one of the duties of the County Council, as the education authority, to ensure medical inspection and treatment of the elementary scholars in its area. There appears to be an impression in some quarters that disease among children is practically confined to the crowded districts of our great towns. Unfortunately that is not the case, and in Surrey a very large number of children, even in the rural areas, have been found to be suffering from, more or less, serious ailments of the eyes, ears, nose, teeth, etc. So far very little has been done to ensure that these defects are remedied. Here and there, through philanthropy or a local hospital, some cases have been attended to; but we have it on the authority of the medical officer that in no less than 58 per cent. of the cases recommended for treatment last year no action at all was taken. To meet this serious position the Education Committee of the Council has now put forward a scheme for the establishment of twelve school clinics, which may ultimately be increased to twenty-four, in various parts of the county, which will entail the engagement of twelve school nurses, six assistant medical officers, and three whole-time dentists. There is every prospect of the scheme being launched and carried to a successful issue.

### BOLINGBROKE HOSPITAL, WANDSWORTH.

This is one of the most useful and needed of Metropolitan hospitals, situated in a crowded district, where the residents are mostly of the artisan class. It has fought an uphill fight with commendable perseverance under the chairmanship of Canon Erskine Clarke and other devoted workers, and should win the sympathy and generous support of a few wealthy people who are interested in hospitals and willing to identify themselves with the work of completing the necessary hospital accommodation for the Metropolis of the Empire. The Bolingbroke Hospital has been placed in a most difficult position by the decision of the Courts not to uphold an agreement in connection with the apportionment of the money left by the late Benjamin Weir, which had received the approval of the Charity Commissioners. This hospital had received £5,000 from the trustees of this will, but on the Courts giving an adverse decision the governors with proper spirit faced the music and decided to repay this large sum to the trustees. This step placed the Bolingbroke Hospital in great financial difficulties, having regard to the urgent necessity for extending the hospital, and the money has not yet been made up to them, as it ought in our opinion to be, by the public without hesitation or delay. £5,000 is a large sum for a poor hospital to give up. It has materially diminished the funds by means of which this hospital has for years past carried on its work. All who are interested in the voluntary hospitals should spare a little time to make themselves familiar with the facts and then resolve to give a little personal service, if they are unable to give money, to help its committee to replenish their treasury. We hope that the situation of this hospital in the centre of a large population of relatively poor people, out of sight as it is of the wealthier classes, may attract attention through the press, and so secure the generous help and personal interest of those willing to lend a hand in due appreciation of its needs. The secretary is Mr. J. Lawrie, and the address Bolingbroke Hospital, Wandsworth, S.W.

### NEW X-RAY INSTALLATION FOR THE SALOP INFIRMARY.

Through the generosity of Mr. and Mrs. McCorquodale, of Cound Hall, Shrewsbury, the Salop Infirmary can now boast of a well-equipped light department, with a thoroughly up-to-date *x*-ray apparatus, Finsen light, and other electrical apparatus. At the opening ceremony on February 8, Mr. Mansell (chairman of the institution) explained how the old *x*-ray plant, which has been in use at the infirmary for fifteen years, had become somewhat obsolete, and the directors were consequently in a dilemma when Mr. and Mrs. McCorquodale stepped into the breach and offered to defray the cost of a new installation, expressing the wish that the best instruments should be got, and that the department should be made as complete and up to date as possible. Some structural alterations were rendered necessary in order to get the fullest advantage of the new installation, and a ward on the ground floor has been partitioned into six compartments, including an observation room, lined by lead sheathing to protect the operator from the rays; a photographic room; a treatment room; the other compartments containing the Finsen light and electrical appliances. A dark room has been built in the basement, and the small *x*-ray apparatus hitherto in use has been moved to the casualty department for the use of the resident medical staff.

### "HELENA" WING AT THE ROYAL FREE HOSPITAL.

At the last quarterly meeting of the committee of management of the Royal Free Hospital it was reported that Princess Christian has promised to lay the foundation-stone of the new out-patient department and extension of the hospital in May next, and that she has consented to the christening of this extension as "Helena" Wing. As for the necessary funds, progress is being made; but an acceleration of the present rate will be necessary if the contingent gift of £5,000 is to be converted into a tangible asset. It will be remembered that this sum was promised on condition that £15,000 was raised by March 4. There was, at the time of the quarterly meeting, still £8,000 needed to make up this sum; but donations to the extent of almost £1,000 were announced, including one of £250 from Sir Edwin Durning Lawrence, who presided at the meeting. Even with this welcome help added there is a large sum still wanted; and those who object to the State confiscating a large slice out of their bequests to charities might well take a leaf out of Mr. Wood's book, and convert any legacies they intend for the Royal Free Hospital into immediate gifts.

### A COLNEY HATCH IMPROVEMENT.

A scheme to provide suitable accommodation for the medical superintendent at Colney Hatch Mental Hospital has been approved by the Asylums Committee of the London County Council. The present quarters are inconvenient and quite unsuitable, more particularly for a married man. The committee point out that a new detached house for the medical superintendent, similar to those provided at the more recent mental hospitals, can be built for a sum not exceeding £2,740. The present quarters are attached to the main building, and in the same block are quarters for the house steward. When the new house is ready for occupation, it is proposed that the steward should be required to live outside the mental hospital (as is the practice at every other London mental hospital) and should be granted a money allowance in lieu of the emolument of a house. The vacated officers' block could then be altered and adapted to provide sleeping accommodation on its second and third floors for 66 patients. Some rooms adjacent on the first floor of the main building, at present used as a head attendants' office and a library, can also be converted into dormitory accommodation for 15 more beds, thus furnishing a total of 81 beds. On the ground floor, which it is proposed to reserve for administrative purposes, quarters would be furnished for an assistant medical officer, with a library to take the place of that converted into patients' accommodation, and inspectors' office, offices for head attendants, and a room for the use of visitors to patients. The male attendants' mess room is to be enlarged, and a new recreation room provided by disusing a number of small rooms, where 16 beds are now available for patients. Rooms for six male attendants will also have to be provided in the wards, and this will reduce the net addition to the total accommodation of the mental hospital to 59 beds. The committee point out that the expenditure, estimated altogether at £4,906, will not only result in very necessary improvements, but, by providing extra accommodation, will mean a saving to the rates of £387 in the first year, and the saving will increase year by year as the debt is paid off.

### PONTYPOOL AND DISTRICT HOSPITAL.

There is a distinguished surgeon and teacher of surgery, now retired, who was noted for the picturesque settings he would give to clinical problems when expounding them to a ward class of medical students. "Now, Mr. Smith," he would begin, addressing some favourite dresser, "this, as you know, is a case of compound fracture of the tibia and fibula. Suppose that you are practising in a colliery district, and that such a case is brought to you, with the whole wound full of earth and coal-dust, with the main artery severed, and with no hospital within forty miles. You will, of course, decide to amputate, and you will have to get the local chemist's assistant to give chloroform, and the mine manager to assist at the operation. All the men who can see through the windows will be watching you; and remember they are keen critics, for they have all seen amputations done before. Your handling of the case will make or mar your future, as far as that neighbourhood is concerned. Now tell us exactly how you will proceed, from the beginning to the end." This was usually a most valuable lesson in how to manage emergency surgery in unfavourable surroundings, impressed upon the minds of the class so enduringly that it was never afterwards forgotten. The state of things thus pictured is gradually passing away; for collieries and other industrial districts are steadily developing the cottage hospital and local hospital system, and the intelligent, usually well-paid, artisans of such thickly populated regions are well content to support these hospitals with liberality.

### A CREDITABLE REPORT.

Let us take the Pontypool and District Hospital, whose report for the past year has just been issued, as an example of what such hospitals can achieve under good management; which is, of course, not the monopoly of the institution in question. A glance through the accounts and the list of governors for 1913 shows at once how thorough is the organisation for securing the financial support of the different classes of workpeople in the locality, and how thoroughly democratic in consequence is the management of the institution. Thus the executive board consists of three representatives of the colliery employees, one of the colliery owners, one of the sheet and steel workers, one of the Pontypool Chamber of Trade, and one of the railway employees. Contributions from employees amount to £1,985; from employers to £458; from collections to £171; while subscriptions from other sources amount to £59. Other items bring the total income to £3,200; whilst the total expenditure is only £2,069. Of this realised surplus, £600 has been invested in the local Gas and Water Company's 4 per Cent. Debenture Stock, and £450 is on deposit with the bank. The policy of building up an investment reserve in good years is a very wise one; and if there is no further coal strike this year there should be an even greater surplus for investment next year. The only point in the accounts that seems not quite admirable is the omission to allow for depreciation of investments. Thus several Consol holdings are treated as if Consols were still at par; the other gilt-edged securities are over-valued in the same way. The work done speaks for itself. Admissions, 508; of which thirty were medical and the rest surgical, including 107 accident cases. Two hundred and eight of the total were children. Three hundred and ninety-one operations were performed, including a good many major abdominal sections. Two hundred cases were treated in the $x$-ray department. Such a record at an expenditure of just over £2,000 shows that the miners get good value for their money.

### WORK IN A METROPOLITAN INFIRMARY.

The work accomplished in Poor-Law infirmaries is exemplified in a return presented to the Bermondsey Board of Guardians by Dr. E. S. Bell, M.R.C.S. (Eng.), M.D., L.R.C.P., the medical superintendent. In twelve months 3,161 patients were admitted to the Bermondsey Infirmary suffering from all sorts and variety of diseases. The proportion of acute to chronic and incurable cases increased, and Dr. Bell estimates the latter to be about 48 per cent. of the total. The discharges and transfers to other institutions numbered 2,083. In the laboratory a large amount of bacterial and pathological work is performed, and nearly forty *post-mortems* were made. Surgical operations numbered 425, excluding a large number of minor operations. The usual nursing lectures and demonstrations were given during the year, and the results of the examinations held for the granting of certificates of proficiency in general nursing were very satisfactory. Dr. Bell pays a tribute to the assistance he has received from Mrs. A. M. Orchard, the matron, and his assistants, Drs. Grean and Stewart.

### TUBERCULOSIS REGULATIONS.

On two or three recent occasions we have noted new developments in the regulations made by the Local Government Board with regard to tuberculosis. It is now, as we have previously announced, compulsory to notify all forms of tuberculosis. There are, however, two conditions in which there is no need to notify the Medical Officer of Health. One is when the diagnosis rests solely upon evidence derived from tuberculosis tests, and not at all from clinical signs or symptoms; the other is when the practitioner has reason to believe that the case has already been notified to the Medical Officer of Health, either of the district where the patient is actually residing or of any other district in which he may have already resided. Under some of the regulations issued in 1911 it was made to appear that institutional officers were eligible for the same half-crown fee for each notification which private practitioners receive. But the new regulations and circulars show quite definitely that this is not the case, and that the scale of fees is exactly the same as for other notifiable diseases. We have frequently alluded to this anomaly and urged its repeal in these columns. In respect of tuberculosis it is further noteworthy that notifications received from medical inspectors of school children and from medical officers of tuberculosis dispensaries and sanatoria are not to be remunerated at all. An alteration which institutional medical officers will do well to take instant cognisance of is the requirement that in-patients whom it is necessary to notify are to be notified to the Medical Officer of Health in the district of their residence before admission, not, as hitherto, to the Medical Officer of Health for the district in which the hospital or institution is situated. Lunatic asylums are now included in the Board's definition of a hospital.

### PRIZES FOR MEDICAL DISCOVERIES.

The coming International Congress of Medicine, to be held in London next summer, is the seventeenth of the series. It has been widely announced and advertised, and there is every reason to hope that it will be entirely successful. It may not be so well known that three valuable prizes are awarded at each Congress, known respectively as the Prize of Moscow, the Prize of Paris, and the Prize of Hungary. The first is of five thousand francs in value, and is awarded for the best work in medicine or hygiene, or for eminent services rendered to suffering humanity. The second is worth four thousand francs, and will be given to a single individual for a discovery or a series of original researches, not more than ten years old, and bearing on medicine, surgery, obstetrics, anatomy, or biology. The third amounts to three thousand crowns, and goes to premiate any work in any medical science which has appeared since the last Congress but one. The last day for sending in nominations is June 1, and apparently anyone may enter his own name for the competition, or that of someone else whom he deems worthy of being honoured. The permanent office of the Congress, to which all such entries must go, is at 10 Hugo de Groot straat, The Hague.

### BATH AND BATHERS.

It is stated that a second medical visit to Bath is proposed, and one that is to be on a larger scale than that recently undertaken, and to include members of the Continental profession. The proposal has been criticised in the West Country on the grounds that members of the local profession and others in a position to judge have declared the bathing arrangements to be incomplete, and in some respects entirely out of date. If this is really the case, it cannot do the town or the reputation of its baths any good to adopt so flamboyant an advertisement. After the recent discovery of marked radioactivity in the waters, it would be a thousand pities that any imperfection of accommodation or administration should mar the impression which foreign visitors are likely to receive. With the general principle of bringing into prominence our British spas we heartily concur. Many of them are intrinsically of far greater curative and therapeutic value than some of the flourishing spas abroad; and lack visitors simply through lack of efficient organisation, accommodation, and advertisement.

### ANOTHER CURIOUS DISCOVERY AT CANE HILL.

In a recent issue of The Hospital it was announced that there had been discovered in the grounds of the Cane Hill Mental Hospital at Coulsdon remains which were supposed to be those of a mammoth and a hippopotamus. Another remarkable discovery has recently been made. In the course of further excavations on the Mental Hospital estate there have been found human remains of early Anglo-Saxon date. It is thought possible that the remains belong to the sixth century, and it is suggested that the estate covers the site of what was, in earliest times, an extensive cemetery.

### SITE CHOSEN FOR THE CARDIFF MEDICAL SCHOOL.

It was decided last week by the Council of the University College of South Wales and Monmouthshire to build the proposed Cardiff Medical School in Newport Road, on the site of the old college. This site has the great advantage of being quite close to King Edward VII.'s Hospital. Mr. W. James Thomas, of Ynyshir, a munificent supporter of the hospital, has increased his gift of ten thousand guineas towards the medical school to twelve thousand guineas.

